# Facile One-Step Sonochemical Synthesis and Photocatalytic Properties of Graphene/Ag_3_PO_4_ Quantum Dots Composites

**DOI:** 10.1186/s11671-018-2466-9

**Published:** 2018-03-02

**Authors:** Abulajiang Reheman, Yalkunjan Tursun, Talifu Dilinuer, Maimaiti Halidan, Kuerbangnisha Kadeer, Abulikemu Abulizi

**Affiliations:** Key Laboratory of Coal Conversion & Chemical Engineering Process (Xinjiang Uyghur Autonomous Region), College of Chemistry and Chemical Engineering, Xinjiang University, Urumqi, 830046 People’s Republic of China

**Keywords:** rGO/Ag_3_PO_4_ QDs composite, Sonochemical method, Photocatalytic stability, Methylene blue

## Abstract

In this study, a novel graphene/Ag_3_PO_4_ quantum dot (rGO/Ag_3_PO_4_ QD) composite was successfully synthesized via a facile one-step photo-ultrasonic-assisted reduction method for the first time. The composites were analyzed by various techniques. According to the obtained results, Ag_3_PO_4_ QDs with a size of 1–4 nm were uniformly dispersed on rGO nanosheets to form rGO/Ag_3_PO_4_ QD composites. The photocatalytic activity of rGO/Ag_3_PO_4_ QD composites was evaluated by the decomposition of methylene blue (MB). Meanwhile, effects of the surfactant dosage and the amount of rGO on the photocatalytic activity were also investigated. It was found that rGO/Ag_3_PO_4_ QDs (W_rGO_:W_composite_ = 2.3%) composite exhibited better photocatalytic activity and stability with degrading 97.5% of MB within 5 min. The improved photocatalytic activities and stabilities were majorly related to the synergistic effect between Ag_3_PO_4_ QDs and rGO with high specific surface area, which gave rise to efficient interfacial transfer of photogenerated electrons and holes on both materials. Moreover, possible formation and photocatalytic mechanisms of rGO/Ag_3_PO_4_ QDs were proposed. The obtained rGO/Ag_3_PO_4_ QDs photocatalysts would have great potentials in sewage treatment and water splitting.

**Electronic supplementary material:**

The online version of this article (10.1186/s11671-018-2466-9) contains supplementary material, which is available to authorized users.

## Background

Recently, synthesis of photocatalysts with high efficiency has captured the attention of the researchers because of their potential applications in the removal of organic pollutants and hydrogen production [1–3]. Because of high activation and efficient separation of photoexcited electrons(e^−^) and holes(h^+^) [4], Ag_3_PO_4_ semiconductor photocatalysts received extensive attention of researchers in the field of photocatalysis. Unfortunately, there are several factors which influence the photocatalytic performance of Ag_3_PO_4_, such as irregular morphology, poor solubility, unstability, high cost, etc., which hindered its widespread applications [5]. Therefore, it is necessary to enhance the photoactivity and photostability of Ag_3_PO_4_.

Previous researches have proved that the photocatalytic performance could be significantly improved by the efficient separation of photogenerated e^−^-h^+^ pairs [6–8]. According to the equation *τ* = *r*^2^/π^2^*D*, where *τ* represents the average diffusion time of the photogenerated carriers, *r* stands for the particle radius, and *D* refers to the carrier diffusion coefficiency [9], reduced particle size may benefit for the efficient suppression of charge carrier recombination, thus improving the photocatalytic activity of the photocatalysts. It can be deduced from this viewpoint that the presence of quantum dots (QDs) could enhance the photocatalytic activity [10, 11]. Because surfactant coverage can hinder the mutual contact between QD surface and pollutants, QDs are seldom reported to be applied as high-efficient photocatalyst independently. In order to supplement this defect, QDs were usually loaded on a carrier with large surface area to decrease the aggregation in the absence of any stabilizer, which endows QDs with the enhanced photocatalytic activity.

Due to better electron separation and transfer in heterostructures, rGO was chosen to be the supporter for the Ag_3_PO_4_ QDs. rGO has a two-dimensional (2D) carbon structure with outstanding electronic, mechanical, and thermal properties [12], high specific surface area, and high carrier mobility [13–16].These properties make it a good substrate for Ag_3_PO_4_ photocatalyst, because it could effectively promote the e^−^-h^+^ pair separation and faciliate the charge transfer between the heterojunctions to improve photocatalytic activity and stability. Furthermore, rGO could be produced by a chemical oxidation and reduction procedure [17]. The methods of graphene oxide (GO) into rGO include chemical vapor deposition (CVD) reduction [18, 19], chemical reduction [20], and hydrothermal reduction [21, 22]. However, the above methods have some intrinsic drawbacks such as complex procedure and secondary pollution. Therefore, it is necessary to develop a green way to produce rGO. Recently, the new green ways of photo-assisted [23, 24] and ultrasonic-assisted [25] reduction method were reported.

Photoreduction of GO to produce rGO is a mild and green method; besides, photochemical and photothermal reduction mechanisms may take place individually or coinstantaneously in the processes [26–28]. Furthermore, the self-photoreduction of GO to rGO can enhance the presence of hole scavenger in the solution [24]. Ultrasound has been widely used for the material synthesis and wastewater treatment [29, 30].Ultrasonic irradiation can offer localized hot spots with pressure about 20 MPa, temperatures about 5000 K, and high cooling rate about 10^10^ Ks ^− 1^, which are generated by acoustic cavitation [31]. Upon the ultrasonic irradiation, a variety of physical and chemical effects can be produced in the liquids by acoustic cavitation, and a unique chemical reactions environment can be provided under these extreme conditions [31, 32].However, to the best of our knowledge, the synthesis of rGO/Ag_3_PO_4_ QD composites using a photo-ultrasonic-assisted reduction method has not been reported yet.

Herein, we report the design and development of rGO/Ag_3_PO_4_ QD composites with high-efficient photocatalytic performance, wherein the Ag_3_PO_4_ QDs with a size of 1–4 nm were loaded uniformly on rGO nanosheets via a facile one-step photo-ultrasonic-assisted reduction method for the first time. The composites were analyzed by various techniques. The photocatalytic activity and stability of the obtained composites were evaluated by the degradation of methyl orange (MO), Rhodamine B (RhB), and methylene blue (MB) under visible light irradiation. Meanwhile, the surfactant dosage and the amount of rGO on the photocatalytic performance were also discussed. The possible photocatalytic mechanism of rGO/Ag_3_PO_4_ QDs was analyzed based on the free radicals trapping experiments. This paper will provide a facile and green method for the fabrication of multiple metal oxide QDs and efficient functional materials with broader application in the field of environmental purification.

## Experimental Section

### Synthesis of rGO/Ag_3_PO_4_ QDs

GO was prepared from natural graphite based on Hummers method [33]. In a typical synthesis process, 20 mg of GO was added in 50 mL of water and sonicated for 30 min to form a uniform suspension, and then 2.2 mmol sodium oleate was added into the above solution and sonicated for 60 min. After that, 10 mL AgNO_3_ aqueous solution(0.6 mol/L)was added, the obtained solution was stirred for 4 h to complete ion exchange, and then 10 mL Na_2_HPO_4_ aqueous solution (0.2 mol/L) was added drop by drop to the solution under ultrasonic irradiation. After 60 min, the precipitate was centrifuged (5000 rpm) for 5 min and washed several times with hexyl alcohol and absolute ethanol to obtain GO/Ag_3_PO_4_ QD composites. Hereafter, 0.3 g of GO/Ag_3_PO_4_QDs was dissolved in 100 mL absolute ethanol, and the mixture was exposed to visible light irradiation (CEL-S500, 300 W Xe lamp, 420 nm cutoff filter) and ultrasonic irradiation for 60 min. The ultrasonic irradiation was performed with a high-intensity ultrasonic probe (Xinzhi Co., China, JY92-2D, 10 mm diameter, Ti-horn, 20 kHz) which was placed in the reaction system. The precipitate was centrifuged (5000 rpm) for 5 min and then dried at 60 °C for 12 h to obtain rGO/Ag_3_PO_4_ QD composites. Ag_3_PO_4_ QDs were prepared under the same condition without GO. To investigate the optimal rGO loading amount, a series of samples with theoretical weight ratios of rGO to rGO/Ag_3_PO_4_ QD composites (W_rGO_:W_composite_ = 1.5, 2.0, 2.3, 2.5 and 3.0 wt%) were obtained. The corresponding rGO/Ag_3_PO_4_ QD composites were marked as R-1.5, R-2, R-2.3, R-2.5, and R-3.

### Materials Characterization

Ag_3_PO_4_ QDs and rGO/Ag_3_PO_4_ QD composites were analyzed by X-ray diffraction (XRD, Cu-Ka, *k* = 1.5418 Å) in 2θ range from 10° to 80°, FT-IR spectroscopy, TEM (JEOL JEM-2010), Raman spectra system (Horiba JY-T64000, France), XPS (PHI Quantera SXM) spectrometer, and UV-vis spectrophotometer (U-3010, Hitachi, Japan). Photoluminescence spectra were obtained by FL (F-4500, Hitachi, Japan) spectrophotometer.

### Photocatalytic Activity Measurement

To measure the photocatalytic properties of the composites, 10 mg of the prepared samples was added to 100 mL of 10 ppm MB. The mixture was magnetically stirred for 30 min under the dark to ensure absorption–desorption equilibrium. A filter (*λ* ≥ 420 nm) was placed on the beaker and then was irradiated with a 300 W xenon light source(CEL-S500, China). In the beginning, the samples were collected in every 1 minute, until 6 min, and then the samples were taken out in every 2 min. A UV-vis spectrophotometer was used to analyze the absorbance properties of the collected solution. The photocatalysts were removed by centrifugation (12,000 rpm, 3 min) before UV-vis measurements.

### Detection of active species

The trapping experiment was conducted in a similar way with the photocatalytic degradation experiment. Three different scavengers including (concentration was about 1 mM) isopropanol (IPA, OH· scavenger), disodium ethylenediaminetetraacetate (EDTA, hole scavenger), and p-benzoquinone (BQ, O_2_·^−^ scavenger) were used, respectively, to investigate the main active species generated in the photodegradation process.

## Results and Discussion

### Materials Characterization

**Fig. 1:**
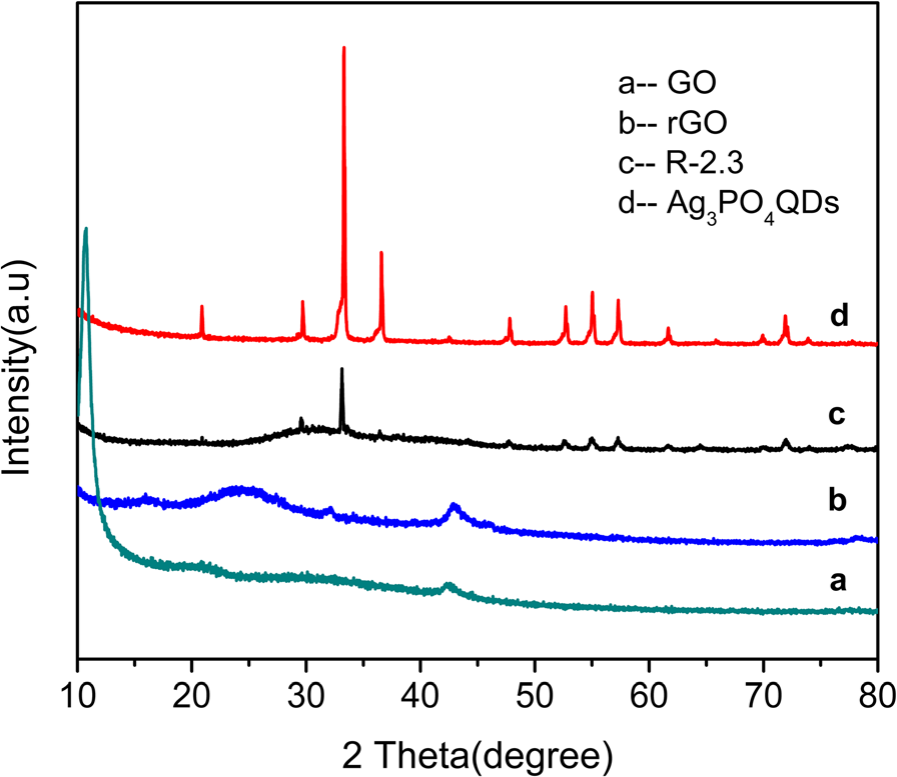
XRD patterns of **a** GO, **b** rGO, **c** R-2.3, and **d** Ag_3_PO_4_ QDs

Figure 1 exhibited the XRD patterns of GO, rGO, Ag_3_PO_4_ QDs, and R-2.3. The XRD results of GO and rGO revealed a characteristic reflection peak at 2θ = 10.7° and 25°, respectively (corresponding to a d-spacing of 0.83, 0.36 nm) (Fig. 1a, b) [34]. All the XRD peaks of Ag_3_PO_4_ can be indexed to the body-centered cubic phase of (JCPDS No.06-0505) (Fig. 1d). The R-2.3 exhibited a similar XRD pattern with pure Ag_3_PO_4_ QDs, and the broader diffraction peaks were attributed to the small size of Ag_3_PO_4_ QDs, which was calculated to be about 3.7 nm according to the Scherrer equation [35]. No diffraction peaks assigned to GO and rGO could be observed in the composites (Fig. 1c), which was attributed to the small rGO amount in the composite [36]. To investigate the effect of GO on the formation of Ag_3_PO_4_ QDs, the XRD pattern of pure Ag_3_PO_4_ QDs was measured. The diffraction peaks of pure Ag_3_PO_4_ QDs could be indexed to cubic Ag_3_PO_4_. The average size of pure Ag_3_PO_4_ QDs was calculated to be about 5.1 nm with the Scherrer equation, which was larger than that of rGO/Ag_3_PO_4_ composites. Above results indicated that GO sheets could affect the formation of Ag_3_PO_4_ QDs.

**Fig. 2:**
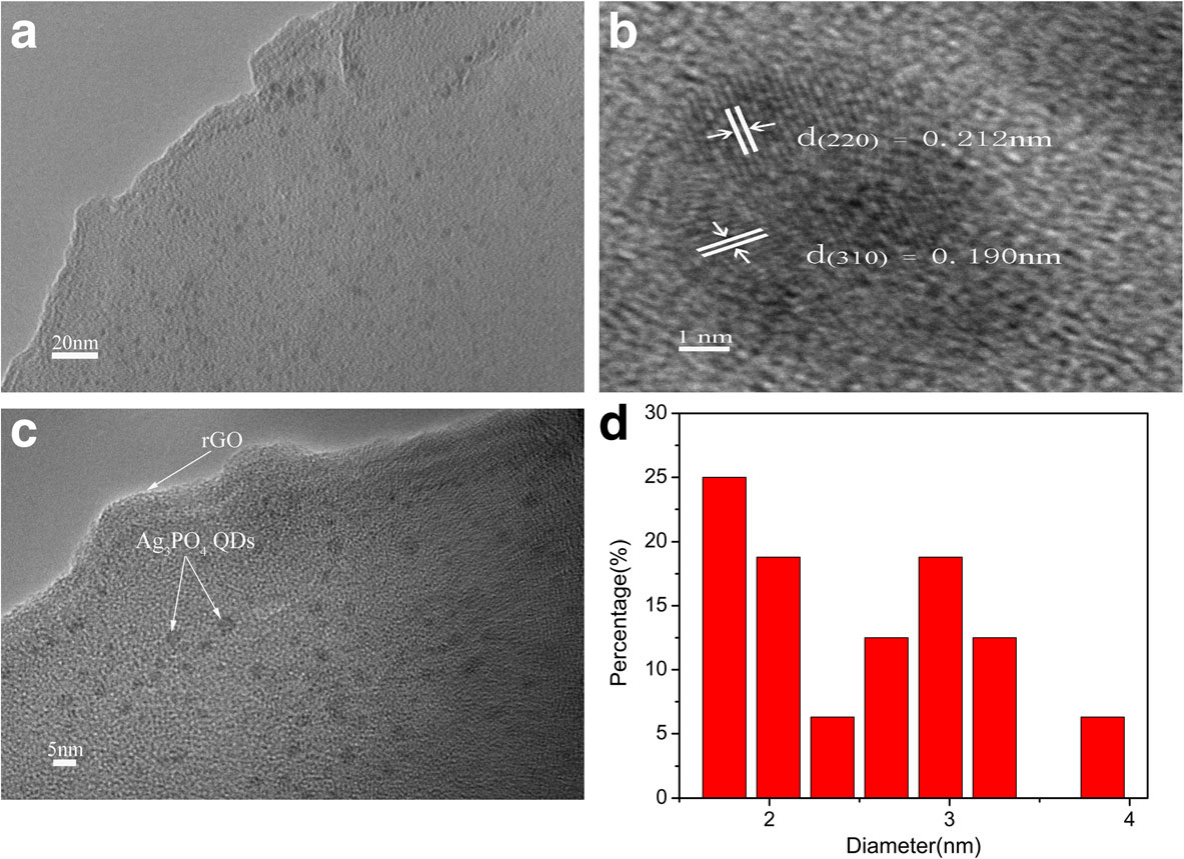
TEM images of R-2.3 (**a**, **c**), HRTEM image of R-2.3 (**b**), and particle size distribution (**d**)

Figure 2 shows the TEM images of R-2.3 composites. Ag_3_PO_4_ QDs which a relatively narrow size distribution with a diameter of 2.81 ± 1.2 nm were dispersed uniformly on rGO sheet. The lattice spacing was 0.212 and 0.190 nm, which corresponded to the d-spacing of (220) and (310) crystallographic plane of Ag_3_PO_4_, respectively. To investigate the effects of ultrasonic, conventional stirring was performed instead of ultrasonic treatment. The results were shown in Additional file 1: Figure S1. Ag_3_PO_4_ particles on rGO which was formed by conventional stirring method did not show uniform structure, and the size of Ag_3_PO_4_ became larger than that formed by ultrasonic treatment. The above results indicated that ultrasonic treatment was very effective in dispersing and controlling size of Ag_3_PO_4_ particles on rGO layers [37].

**Fig. 3:**
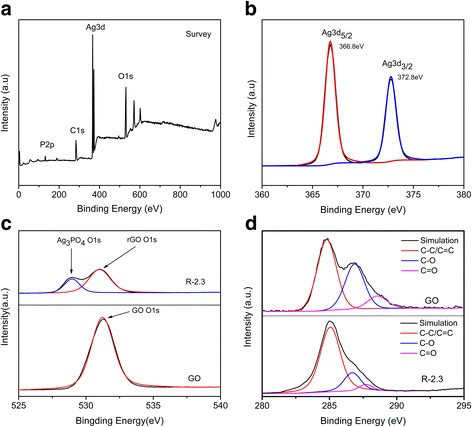
XPS of **a** survey spectrum, **b** Ag3d, **c** O1s, and **d** C1s of GO and R-2.3

The successful ultrasonic-assisted photo-reduction of GO to rGO can be further confirmed by XPS spectra of GO and R-2.3 composites as shown in Fig. 3. The peaks located at 131.7, 284.2, 367.2, and 530.2 eV were indexed to the characteristic peaks of P2p, C1s, Ag3d, and O1s, respectively (Fig. 3a). The strong peaks at 366.8 and 372.8 eV are attributed to Ag^+^ of Ag_3_PO_4_ [38] (Fig. 3b). The O1s XPS spectra of R-2.3 can be divided into two peaks, which were attributed to O1s from Ag_3_PO_4_ (529.5 eV) and O1s from rGO (531.3 eV) [7, 39]. The peak of O1s from rGO (531.3 eV) shifted to lower binding energy compared with that of GO (531.8 eV), implying that there existed a chemical interaction between rGO and Ag_3_PO_4_ QDs by C=O bond. The C1s spectrum of GO was divided into three different peaks at 284.8, 286.7, and 287.7 eV, which were assigned to C-C/C=C, C-O, and C=O, respectively [40, 41] (Fig. 3c). After being reduced by visible light assisted with ultrasonic irradiation (Fig. 3d), the oxygen-containing groups, especially C-O, C=O showed remarkably decreased peak intensities, indicating that the reduction from GO to rGO proceeded successfully.

**Fig. 4:**
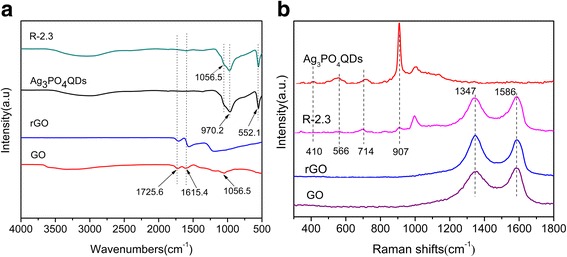
FT-IR spectra (**a**) and Raman spectra (**b**) of GO, rGO, Ag_3_PO_4_ QDs, and R-2.3

Figure 4a demonstrated the FTIR spectra of GO, rGO, Ag_3_PO_4_ QDs, and R-2.3. The characteristic peaks at 1725.6, 1056.5, and 1615.4 cm^− 1^ in GO were attributed to the stretching vibrations of carboxyl C=O, alkoxy C-O, and C=C [40, 42], respectively. The broad peak at 3000–3600 cm^− 1^ was ascribed to the O-H stretching vibration [43]. Ag_3_PO_4_ QDs and R-2.3 composites had similar FT-IR peaks at 552.1 and 970.2 cm^− 1^, which were assigned to vibrations of P-O from PO_4_^3−^ [44]. This indicated that Ag_3_PO_4_ QDs were bonded on rGO sheets. After photo-ultrasonic-assisted reduction to rGO, the characteristic peaks (at 1725.6, 1056.5 cm^− 1^) shifted to lower wavenumbers compared to GO, which was consistent with the results of XPS analysis, indicating the existence of charge interaction between rGO and Ag_3_PO_4_ in the as-prepared composites.

Figure 4b showed the Raman spectra of GO, rGO, Ag_3_PO_4_ QDs, and R-2.3. The Raman spectrum of GO showed two characteristic peaks of the D band at 1347 cm^− 1^ and G band at 1586 cm^− 1^. The value of *I*_D_/*I*_G_ in R-2.3 and in GO was about 1.039 and 0.9056, respectively. It was obvious that the composite showed relatively high intensity of the D band compared with GO, which confirmed that the GO sheets were partially reduced into rGO [37]. The Raman spectra of Ag_3_PO_4_ QDs and R-2.3 showed three distinct peaks at 410, 566, and 714 cm^− 1^, and these peaks were accredited to the P-O-P bonds. The strong peak at 907 cm^− 1^ was raised from the motion of terminal oxygen bond vibration in phosphate chains [23].

### Preparation mechanism of rGO/Ag_3_PO_4_ QDs

**Fig. 5:**
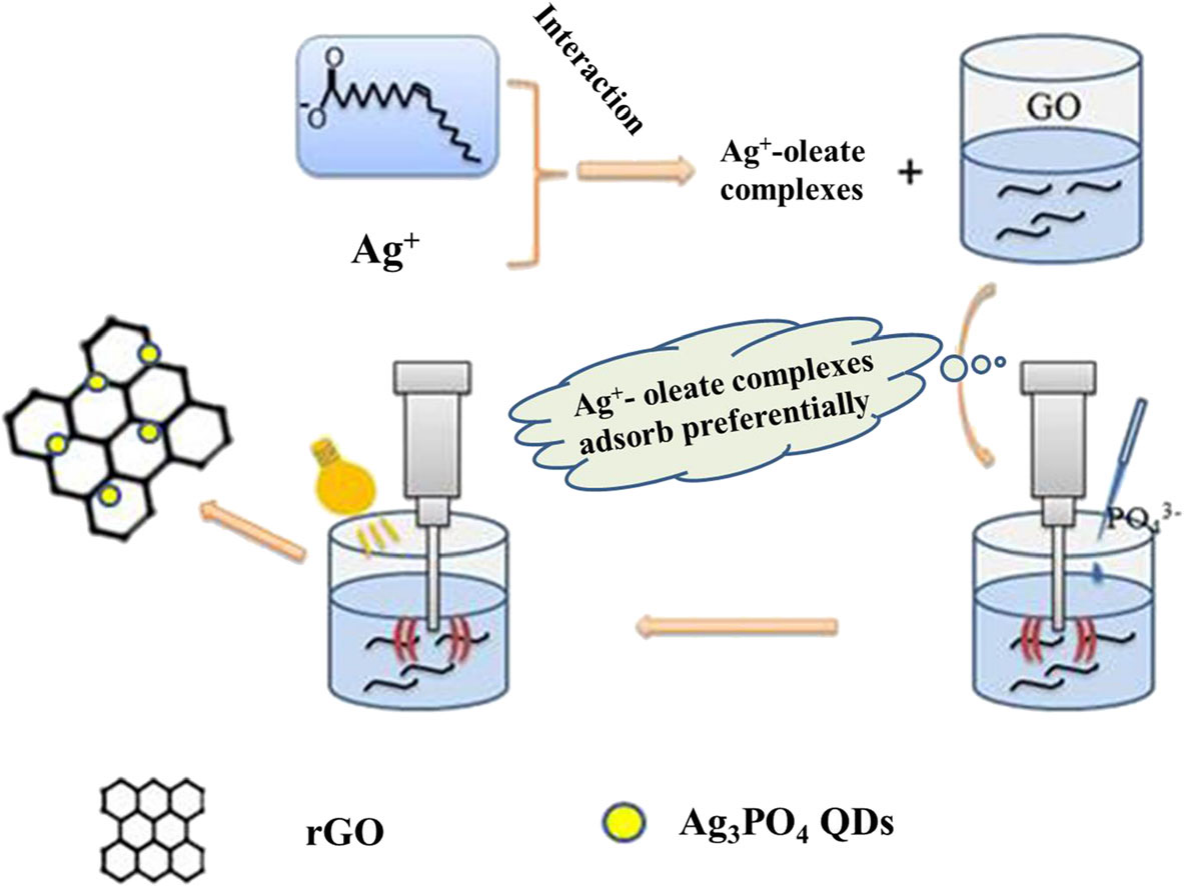
Illustration of the synthesis of rGO/Ag_3_PO_4_ QD composites via photo-ultrasonic-assisted method

The synthesis route of rGO/Ag_3_PO_4_ QD composite was proposed and schematically illustrated in Fig. 5. The synthesis reactions were detailed as follows:

$$ {\mathrm{Ag}}^{+}+\mathrm{oleate}\ \mathrm{ions}\to \mathrm{Ag}\hbox{-} \mathrm{oleate} $$

1 $$ \mathrm{Ag}\hbox{-} \mathrm{oleate}+\mathrm{GO}\to \mathrm{GO}\hbox{-} \mathrm{Ag}\hbox{-} \mathrm{oleate} $$

2 $$ \mathrm{GO}\hbox{-} \mathrm{Ag}\hbox{-} \mathrm{oleate}+{{\mathrm{PO}}_4}^{3\hbox{-}}\to \mathrm{GO}\hbox{-} {\mathrm{Ag}}_3{\mathrm{PO}}_4\mathrm{QDs} $$

3 $$ \mathrm{GO}\hbox{-} {\mathrm{Ag}}_3{\mathrm{PO}}_4+\mathrm{h}\upsilon \to \mathrm{GO}\hbox{-} {\mathrm{Ag}}_3{\mathrm{PO}}_4\mathrm{QDs}\left({\mathrm{e}}^{\hbox{-} }+{\mathrm{h}}^{+}\right) $$

$$ {\mathrm{H}}_2\mathrm{O}\to \cdotp \mathrm{OH}+\cdotp \mathrm{H} $$

$$ \cdotp \mathrm{OH}+\cdotp \mathrm{OH}\to {\mathrm{H}}_2{\mathrm{O}}_2 $$

4 $$ \mathrm{GO}\hbox{-} {\mathrm{Ag}}_3{\mathrm{PO}}_4\mathrm{QDs}+{\mathrm{e}}^{\hbox{-}}\to \mathrm{rGO}\hbox{-} {\mathrm{Ag}}_3{\mathrm{PO}}_4\mathrm{QDs} $$

$$ \mathrm{GO}\hbox{-} {\mathrm{Ag}}_3{\mathrm{PO}}_4\mathrm{QDs}+\cdotp \mathrm{H}\to \mathrm{rGO}\hbox{-} {\mathrm{Ag}}_3{\mathrm{PO}}_4\mathrm{QDs} $$

The total synthesis route could be fallen into four successive stages. Firstly, Ag^+^ and oleate ions interacted electrostatically to form Ag-oleate complexes, hydrolysis of Ag^+^ ions could be prevented effectively by this process. Ag-oleate complexes interacted with the excess of oleate ions improving its hydrophilic property in order to disperse in water. Oxygen groups on the surface of GO provided hydrophilic property. When GO sheets were added to the Ag- oleate aqueous solution, the Ag-oleate complexes will preferentially adsorb on these oxygen containing functional groups (Eq. (1)). Secondly, reactions between Ag^+^ and PO_4_^3−^ proceeded to form Ag_3_PO_4_ QDs on GO surface (Eq. (2)). Thirdly, when GO-Ag_3_PO_4_ QDs were sonicated in solution, the ultrasonic stimulated electron–hole pairs from Ag_3_PO_4_ QDs when GO-Ag_3_PO_4_QDs was irradiated with visible-light in ethanol solution. At the same time, ·H and H_2_O_2_ were produced by ultrasonic irradiation. Ultimately, GO was reduced into rGO by ·H and accepted photo-generated electrons from the conduction band (CB) of Ag_3_PO_4_. As a result, rGO/Ag_3_PO_4_ QD composite was obtained by photo-ultrasonic-assisted reduction.

### Optical properties of photocatalysis

**Fig. 6:**
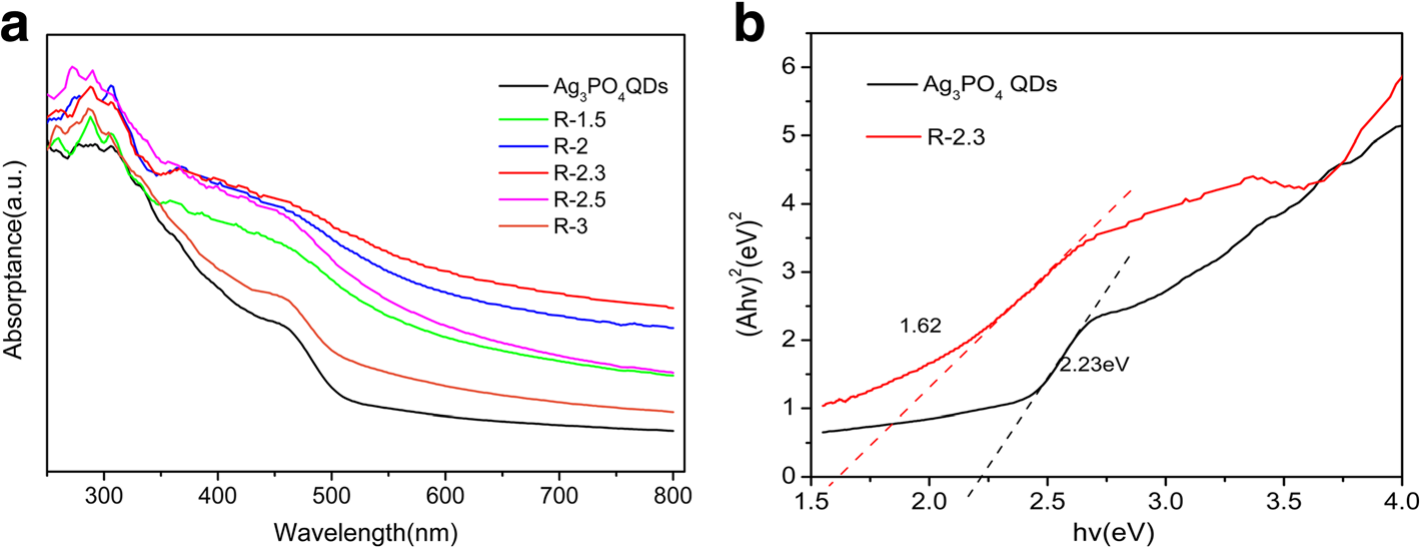
**a** UV-vis DRS spectra of Ag_3_PO_4_ QDs, R-1.5, R-2, R-2.3, R-2.5, R-3 composites, and **b** the plots of (αhν)^2^ versus Eg

The UV-vis absorption spectra of Ag_3_PO_4_ QDs and rGO/Ag_3_PO_4_ QDs with different mass ratio of rGO were shown in Fig. 6a. The absorbance wavelength of pure Ag_3_PO_4_ QDs was shorter than 530 nm; inversely, rGO/Ag_3_PO_4_ QD composites structure showed an extended wavelength (> 530 nm) and its intensity increased with increasing rGO contents before which reached 2.3%, and decreased after. This can be attributed to that the presence of carbon in rGO/Ag_3_PO_4_ QDs reduces the reflection of light [45]. According to the Kubelka–Munk function [46], we can get the band gaps of the photocatalysts as shown in Fig. 6b and Additional file 1: Figure S2; the band gap of R-2.3 was calculated to be 1.62 eV, which was lower than pure Ag_3_PO_4_ QDs (2.23 eV). The relative narrow band gap energy may be attributed to the synergetic effect that sum of the total effect is superior to the single effect after different types of dispersion to interact between rGO and Ag_3_PO_4_ QDs [47], which lead to improve the solar spectrum utilization efficiency of the photocatalysts [36].

### Photocatalytic activity and stability

**Fig. 7:**
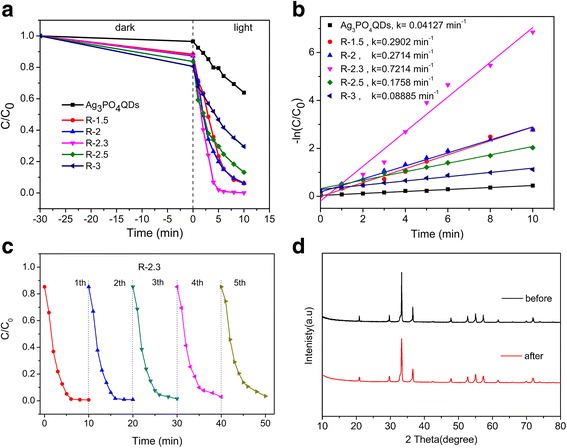
**a** Photocatalytic degradation of MB by Ag_3_PO_4_ QDs, R-1.5, R-2, R-2.3, R-2.5, and R-3 composites, **b** corresponding rate constants (k) of samples for photocatalytic degradation of MB, **c** recycling experiments of the R-2.3 for degradation of MB, and **d** XRD patterns before and after the recycling experiments

To understand the influencing factors on the experimental process to the photocatalytic activity, the effects of different mass of surfactant were investigated as shown in Additional file 1: Figure S3. Samples were prepared when other conditions remained constant. The result showed that the photocatalytic activities increased with increasing the mass of surfactant but decreased after more than 0.5 g, as shown in Additional file 1: Figure S3, which may be ascribed to the excessive oleate ions that limited Ag_3_PO_4_ QDs size distribution on rGO surface [35]. This leads to the decrease of photocatalytic activities. Compared with pure Ag_3_PO_4_ QDs, the concentration of MB decreased rapidly for rGO/Ag_3_PO_4_ QD composites (Fig. 7a). This result indicated that the photocatalytic reaction was related to the existence of active sites [48, 49]. When the content of rGO was 2.3%, the highest photocatalytic activity was emerged and could degrade MB by 97.46% for 5 min. This can be attributed to rGO-semiconductor heterojunction, which had effectively availed the transfer of charge from rGO nanosheets under visible light irradiation [23]. Under the same conditions, when increasing the content of rGO to 3%, the results had proved fact that excessive loading of rGO could reduce the dye and photon absorption on Ag_3_PO_4_ [23]. Importantly, rGO/Ag_3_PO_4_ QD composites displayed superior photocatalytic performance than pure Ag_3_PO_4_ QDs and rGO-based Ag_3_PO_4_ composites [23, 50]. The photoexited electrons(e^−^) could transfer from the CB of Ag_3_PO_4_ QDs to rGO, and rGO in the composites could act as a highway for electron transfer to suppress the e^−^-h^+^ recombination, which accounted for the remarkably improved photo-conversion efficiency [51]. Moreover, interfacial charge transfer could be faciliated due to the larger superfacial area of rGO [52, 53]. On top of that, the photocatalytic degradation efficiency of R-2.3 composite over different organic dyes was investigated as shown in Additional file 1: Figure S4.

To test the stability of the R-2.3 composite, the cycling experiments of the composite for MB were performed (Fig. 7c). The results revealed that R-2.3 composites exemplified higher photocatalytic stability after five cycles, with maintaining its degradation efficiency up to 90%, indicating the good photocayalytic stability. And this may be benefited from the efficient photo-generated e^−^-h^+^ separation. Moreover, the XRD pattern of R-2.3, which was used for five cycles is shown in Fig. 7d, and no obvious peak about Ag is observed this may be attributed to that rGO could facilitate the electron transfer to Ag_3_PO_4_ QDs and decreased the photocorrosion of Ag_3_PO_4_ QDs [23].

### Mechanism of the enhanced photocatalytic performance

**Fig. 8:**
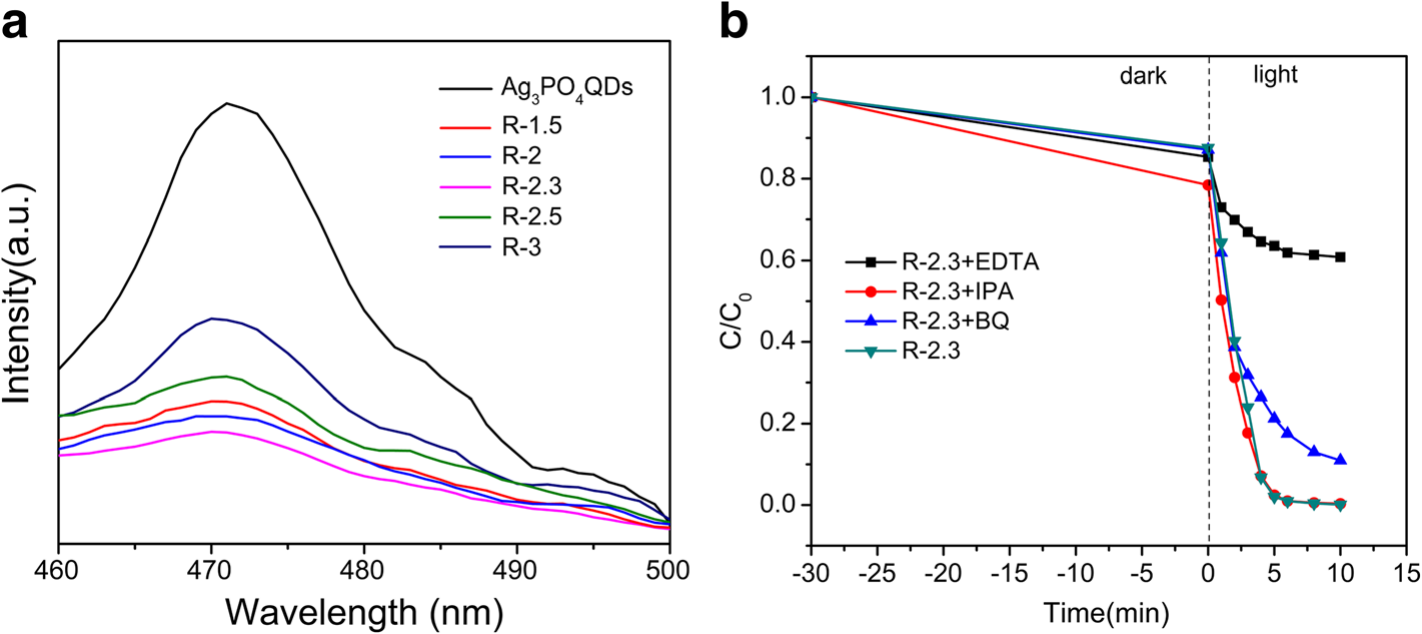
**a** Photoluminescence spectra of Ag_3_PO_4_ QDs, R-1.5, R-2, R-2.3, R-2.5, and R-3 and **b** the effect of different quenchers on the photocatalytic degradation of MB

The aforementioned experimental results indicated that the photocatalytic performance of Ag_3_PO_4_ was enhanced by combining Ag_3_PO_4_ with rGO sheets, which was ascribed to the fast transfer and separation of photo-generated e^−^-h^+^ pairs in the composites [23]. The photoluminescence (PL) spectra were performed to investigate the e^−^-h^+^ pairs migration, transfer, and recombination processes in semiconductors [54, 55]. Figure 8a showed the PL spectra of the samples. The PL spectra of rGO/Ag3PO4 QDs showed a lower recombination rate of photogenerated e^−^-h^+^ pairs compared to Ag_3_PO_4_ QDs, indicating that more photogenerated e^−^ and h^+^ can participate in the reduction and oxidation reaction; this could lead to decline of the recombination of photogenerated e^−^-h^+^ pairs in Ag_3_PO_4_ in the composites. Therefore, rGO/Ag_3_PO_4_ QD composite displayed superior photocatalytic activity than that of Ag_3_PO_4_ QDs.

To further confirm the main active species in the photocatalysis process over rGO/Ag_3_PO_4_ QDs, MB was used as a pollutant. The results are shown in Fig. 8b. Herein, after the addition of isopropanol (as hydroxyl radical scavenger) [56], the catalytic activity of rGO/Ag_3_PO_4_ QDs was not obviously affected; when EDTA (as hole capture) [57] was added, the photocatalytic degradation of MB was greatly inhibited. However, when p-benzoquinone (BQ, O_2_·^−^ scavenger) was added, the deactivation of rGO/Ag_3_PO_4_ QDs was unneglectable. The above results illustrated that holes and O_2_·^−^ radicals were the main active species in the photocatalysis process.

**Fig. 9:**
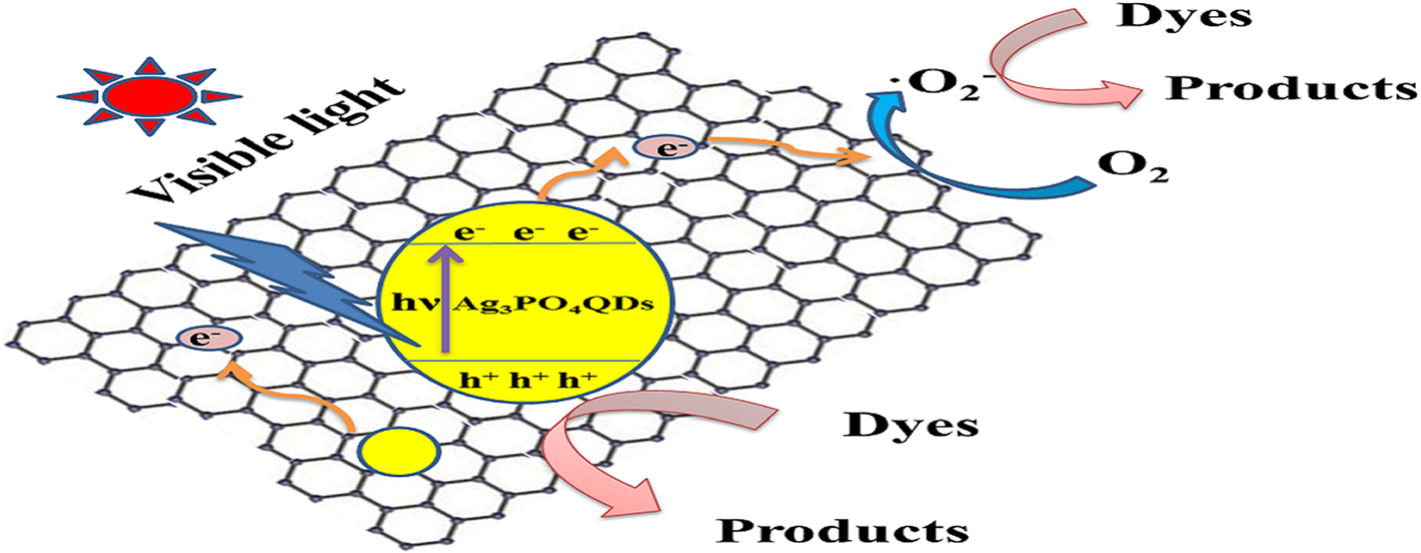
The mechanism for the photocatalytic degradation of organic dyes on the surface of rGO/Ag_3_PO_4_ QD composites

The mechanism for the photocatalytic degradation of organic dyes by rGO/Ag_3_PO_4_ QDs is shown in Fig. 9. Upon the visible light exposure, Ag_3_PO_4_ QDs was photoexcited, and electrons were excited from valence band to conduction band; after that the electrons could transfer to rGO due to effect of the electric field, and then electrons retransferred to the surface of rGO to participate in the photocatalytic reaction. rGO could efficiently separate e^−^-h^+^ pairs, thus availed the transfer of the electrons [23] and led to the promoted photocatalytic activity of rGO/Ag_3_PO_4_ QD composites.

## Conclusions

A novel rGO/Ag_3_PO_4_ QD composite was prepared via a facile photo-ultrasonic-assisted reduction method. The obtained rGO/Ag_3_PO_4_ QD composites exhibited better photocatalytic activity under visible light and higher than pure Ag_3_PO_4_ QDs alone. This was due to the efficient e^−^-h^+^ pairs separation and fast electron transfer in these heterojunctions. The rGO sheets effectively promoted the separation of e^−^ and h^+^ and fast transfer of electrons in the heterostructure photocatalysts. Free radicals trapping experiments indicated that h^+^ played important roles in the photocatalytic degradation of dyes. It was clear that ultrasonic-assisted method was a facile and economical way to prepare visible-light-responsive and high efficient Ag_3_PO_4_ QDs-based composites.

## Additional file

Additional file 1: **Figure S1.** TEM images of rGO/Ag_3_PO_4_ QDs (stirring method). **Figure S2.** The plots of (αhν)^2^ versus Eg of Ag_3_PO_4_ QDs, R-1.5, R-2, R-2.3, R-2.5, and R-3. **Figure S3.** (a) Photocatalyticdegradation of MB by R-2.3 prepared by different mass of surfactant and (b) apparent rate constants (k) of samples for photocatalytic degradation of MB. **Figure S4.** (a) Photocatalytic degradation of MB, MO, and RhB byR-2.3, (b) apparent rate constants (k) of sample for photocatalytic degradation of dyes. (ZIP 12230 kb)

## Abbreviations

2D: Two-dimensional
BQ: p-benzoquinone
CB: Conduction band
CVD: Chemical vapor deposition
EDTA: Disodium ethylenediaminetetraacetate
GO: Graphene oxide
IPA: Isopropanol
MB: Methylene blue
MO: Methyl orange
QDs: Quantum dots
R-1.5, R-2, R-2.3, R-2.5, and R-3: Content of rGO in composites 1.5, 2.0, 2.3, 2.5, and 3.0 wt%
rGO: Graphene
RhB: Rhodamine B
W_composite_: Weight of composites
W_rGO_: Weight of grapheme
