# Supplementary material for: Facile One-Step Sonochemical Synthesis and Photocatalytic Properties of Graphene/Ag3PO4 Quantum Dots Composites
Source: Nanoscale Res Lett. 2018 Mar 2;13:70. doi: 10.1186/s11671-018-2466-9 (PMC5834413; doi:10.1186/s11671-018-2466-9)
Supplement: Supplementary file 1 — Figure S1. TEM images of rGO/Ag3PO4 QDs (stirring method). Figure S2. The plots of (αhν)2 versus Eg of Ag3PO4 QDs, R-1.5, R-2, R-2.3, R-2.5, and R-3. Figure S3. (a) Photocatalyticdegradation of MB by R-2.3 prepared by different mass of surfactant and (b) apparent rate constants (k) of samples for photocatalytic degradation of MB. Figure S4. (a) Photocatalytic degradation of MB, MO, and RhB byR-2.3, (b) apparent rate constants (k) of sample for photocatalytic degradation of dyes. (ZIP 12230 kb) [file 11671_2018_2466_MOESM1_ESM.zip › Supplementary Material.docx]

**Supplementary Material**

**Facile One-step sonochemicalsynthesis and photocatalytic properties of Graphene /Ag_3_PO_4_ quantum dots composites**

**AbulajiangReheman, YalkunjanTursun, TalifuDilinuer, MaimaitiHalidan, AbulikemuAbulizi^[[1]](#footnote-1)^***

**Key Laboratory of Coal Conversion & Chemical Engineering Process (Xinjiang Uyghur Autonomous Region), College of Chemistry and Chemical Engineering, Xinjiang University, Urumqi 830046, PR China.**

^[[2]](#footnote-2)^***Corresponding author:** AbulikemuAbulizi Tel.: +86 13639966149,

Fax: +86-0991-8582078, E-mail address:aabek@163.com, [aablek@xju.edu.cn](mailto:aablek@xju.edu.cn) (A. Abulikemu)

1. **effects of stirring method**

**
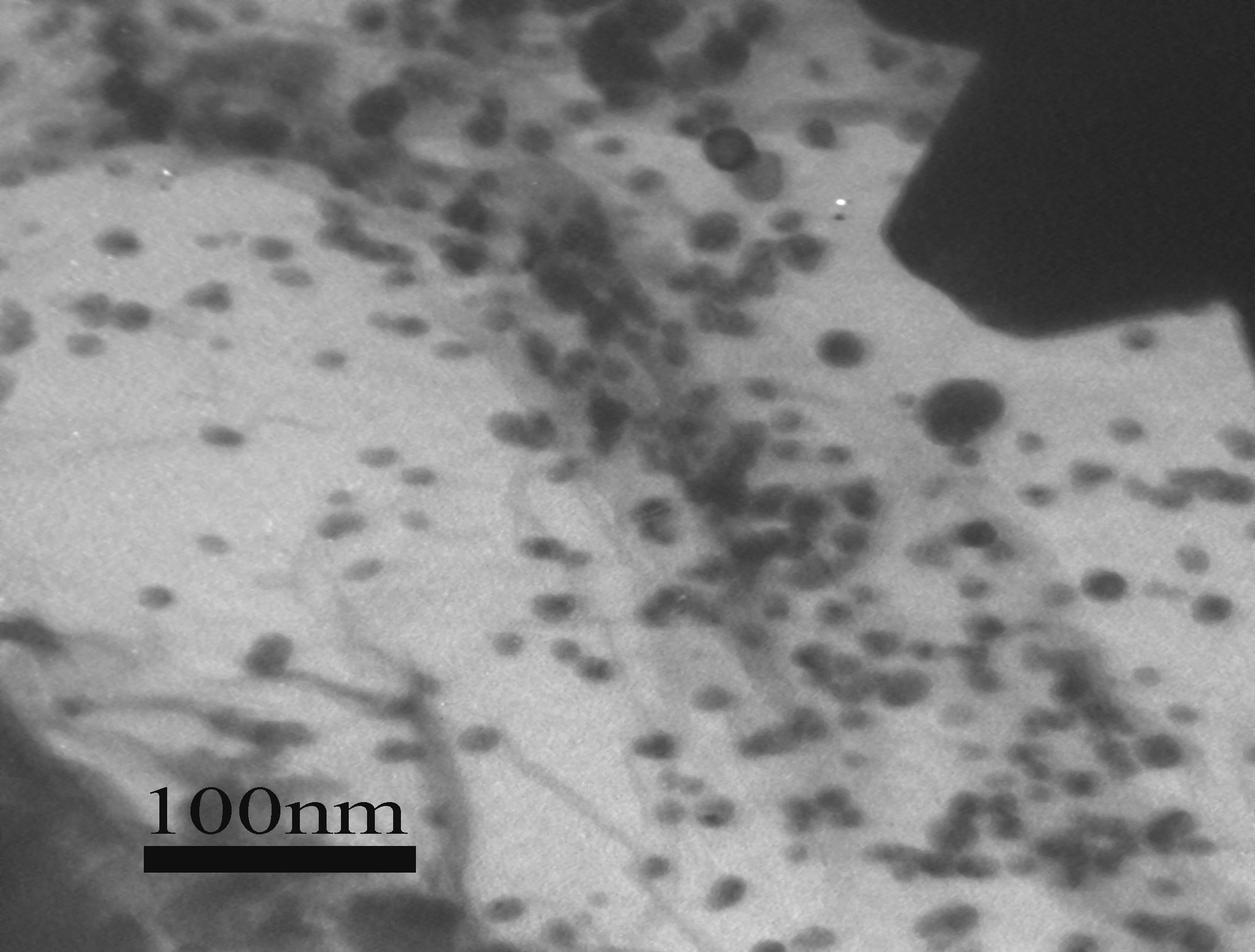
**

Fig.S1 TEM imiges of rGO/Ag_3_PO_4_ QDs (stirring mathod)

**2. UV-vis diffuse reflectance spectrum**

According to the Kubelka–Munk function [[35](#_ENREF_35)],we can get the band gaps of the photocatalysts as shown in Fig.S2，the band gap are about 2.23, 1.83, 1.80, 1.62, 1.84 and 2.19eV for Ag_3_PO_4_ QDs,R-1.5,R-2,R-2.3,R-2.5 and R-3,respectively.





Fig.S2 The plots of (αhν )^2^ versus Eg of Ag_3_PO_4_ QDs,R-1.5,R-2,R-2.3,R-2.5 and R-3.

**3. Effects of surfactant on preparation of rGO/Ag_3_PO_4_ QDs to the photodegrdation**

The photocatalytic activities of the rGO/Ag_3_PO_4_ QDs composites prepared under different mass of surfactant and different pH value were investigated for the degradation of MB. Under visible light irradiation for 6 min, 88.11%(0.1g), 95.46%(0.3g), 98.56%(0.5g), 92.07%(7g) and 80.63%(0.9g) of the initial MB dyes were decomposed , respectively, as shows in Fig.S3.


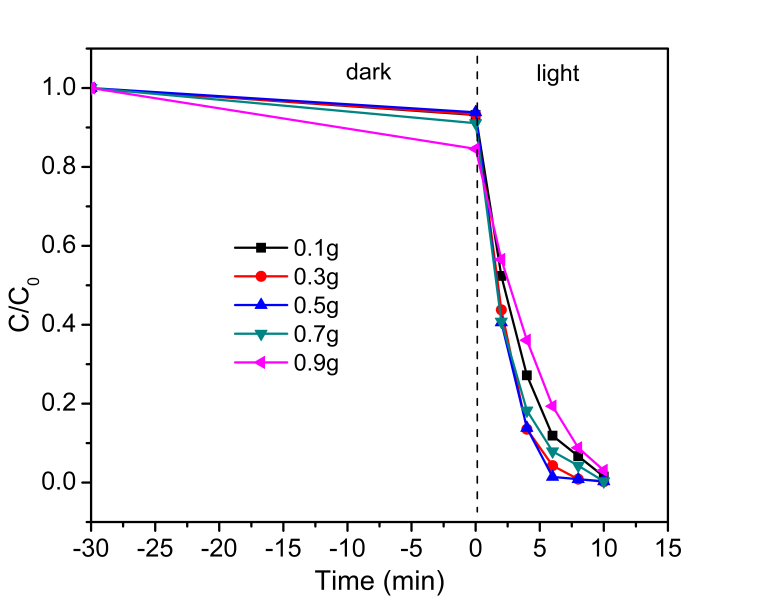

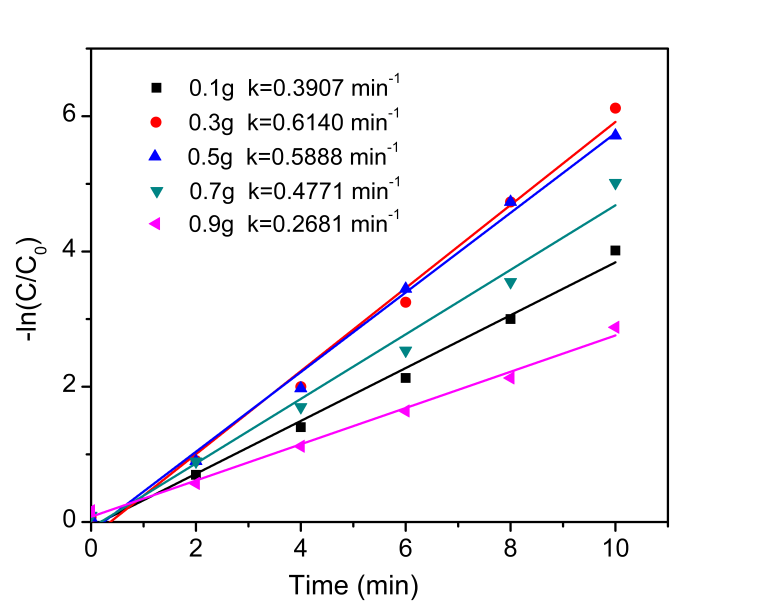


Fig.S3 (a) Photocatalyticdegradation of MB by R-2.3 prepared by different mass of surfactant and (b) apparent rate constants (k) of samples for photocatalytic degradation of MB.

**4. Photodegrdation of R-2.3 for deffrent dyes**

In order to test the extensive applicability, the photodegradation of RhB, MB and MO was investigated by the R-2.3 composite under visible light irradiation.53.73%、97.46% and 20.89% of MO,MB and RhB were decomposed with light irradiation time for 5 min(Fig .S4).







Fig.S4 (a) Photocatalytic degradation of MB、MO and RhB byR-2.3,(b) apparent rate constants (k) of sample for photocatalytic degradation of dyes,

1. [↑](#footnote-ref-1)
2. [↑](#footnote-ref-2)
