# Supplementary figures and images for: Facile One-Step Sonochemical Synthesis and Photocatalytic Properties of Graphene/Ag3PO4 Quantum Dots Composites
Source: Nanoscale Res Lett. 2018 Mar 2;13:70. doi: 10.1186/s11671-018-2466-9 (PMC5834413; doi:10.1186/s11671-018-2466-9)

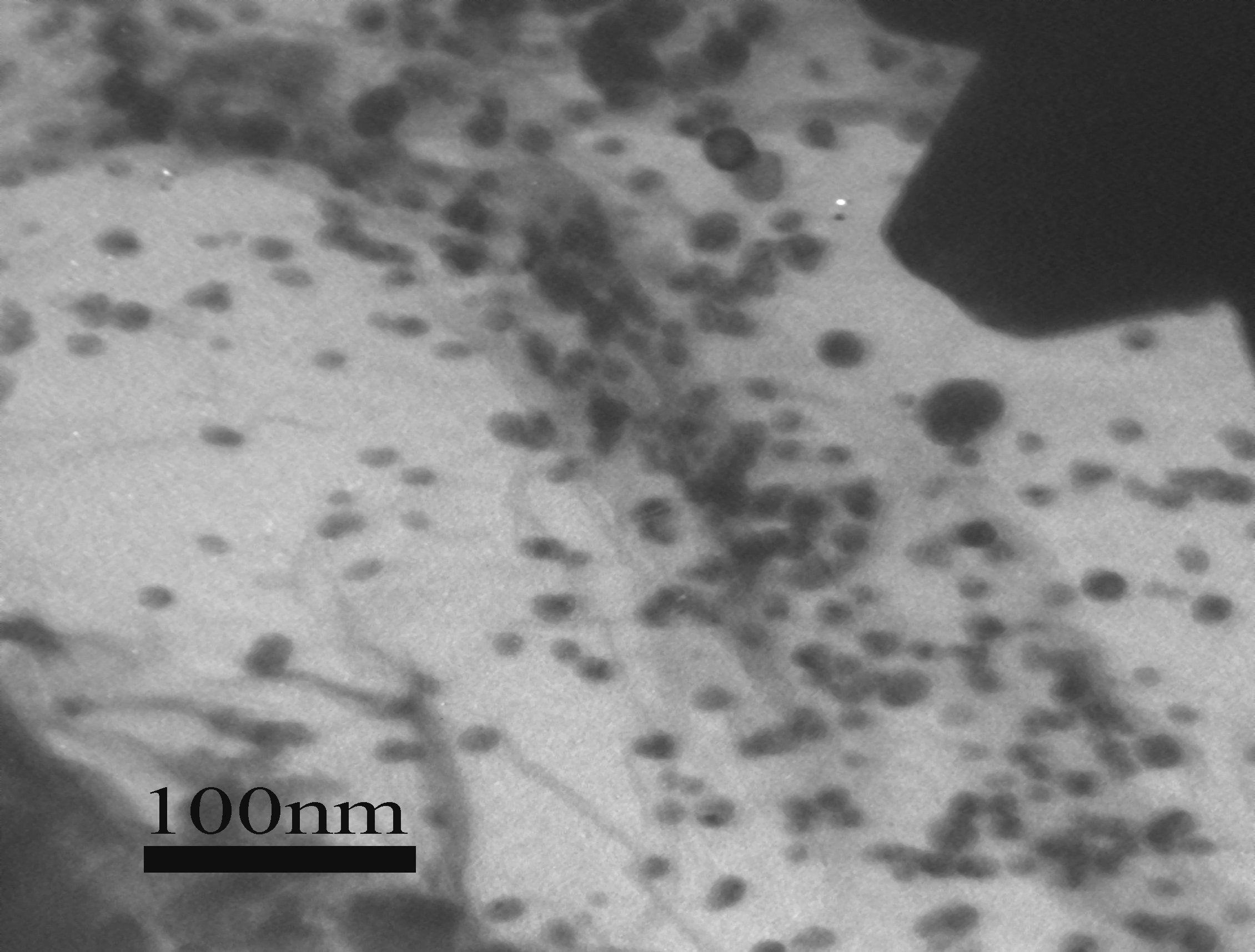

Supplement: Supplementary file 1 — Figure S1. TEM images of rGO/Ag3PO4 QDs (stirring method). Figure S2. The plots of (αhν)2 versus Eg of Ag3PO4 QDs, R-1.5, R-2, R-2.3, R-2.5, and R-3. Figure S3. (a) Photocatalyticdegradation of MB by R-2.3 prepared by different mass of surfactant and (b) apparent rate constants (k) of samples for photocatalytic degradation of MB. Figure S4. (a) Photocatalytic degradation of MB, MO, and RhB byR-2.3, (b) apparent rate constants (k) of sample for photocatalytic degradation of dyes. (ZIP 12230 kb) [file 11671_2018_2466_MOESM1_ESM.zip › Fig.S1.tif]
